# Retooling National TB Control Programmes (NTPs) with New Diagnostics: The NTP Perspective

**DOI:** 10.1371/journal.pone.0011649

**Published:** 2010-07-19

**Authors:** Sanne C. van Kampen, Andrew R. Ramsay, Richard M. Anthony, Paul R. Klatser

**Affiliations:** 1 UNICEF/UNDP/World Bank/WHO Special Programme for Research and Training in Tropical Diseases (TDR), World Health Organization, Geneva, Switzerland; 2 Royal Tropical Institute (KIT), KIT Biomedical Research, Amsterdam, The Netherlands/Athena Institute, Free University of Amsterdam, The Netherlands; University of Stellenbosch, South Africa

## Abstract

**Background:**

A delay is evident between the development of new policies on TB diagnostics and their implementation at country level. The Stop TB Partnership would benefit from information from national TB program (NTP) managers on progress towards implementation of new recommendations as well as the opportunities and challenges encountered in the process.

**Methods and Findings:**

To solicit information on the introduction of new TB diagnostics at country level, questionnaires were sent out to NTP managers of high-burden TB countries and a subset of managers was interviewed. The results indicate that about 50% of high-burden TB countries are using the TB diagnostic tools newly recommended by the World Health Organization (WHO). Most NTP managers reported that new diagnostics would only be implemented when officially endorsed by the WHO. All countries have plans to adopt newly endorsed diagnostics at reference laboratory level, while approaches to optimize smear microscopy at lower levels of the health service are given less attention. NTP managers reported diverse challenges to the implementation of new diagnostics.

**Conclusions:**

More information on the obstacles and advantages of introducing new diagnostic tools should be provided to NTP managers to ensure the rational adoption of new diagnostics. A single recommendation covering the introduction of a package of diagnostic tools might be preferable to NTP managers and facilitate implementation in high-burden TB countries.

## Introduction

Tuberculosis (TB) is a leading global cause of morbidity and mortality and control efforts have so far failed to substantially reduce the burden of disease. Early case detection is critical to TB control but has been problematic, at least partly, because of limitations associated with diagnostic tools. Inadequate diagnostic tools have also contributed to poor detection and poor control of multi-drug resistant TB (MDR-TB) [1]. New diagnostic tools, including new technologies and approaches, are becoming available and are being endorsed by the World Health Organization (WHO). National TB control programmes (NTPs) are beginning to introduce these new diagnostic tools in their diagnostic services and integrate them in national control activities - a process referred to as “retooling”. Diagnostic retooling for control of other infectious diseases has often been slow and significant delays can occur between tools becoming available and their application in control activities. In 2006, the Stop TB Partnership created a Task Force on Retooling to produce a roadmap and other information to facilitate the process and to help reduce this delay [2]. However, anecdotal information suggests that there is still a time gap between WHO-endorsement of new TB diagnostics and their adoption by NTPs. Identifying and understanding this time gap is essential for successful diagnostic retooling.

An audit was conducted in mid-2009 to assess progress in the first phase of retooling, namely adopting new diagnostics by NTPs in countries with a high burden of TB (HBCs) and/or a high-burden of MDR-TB (HBC-MDR) [3]. This aimed to determine whether delays were occurring and if so, to identify barriers to retooling diagnostic services.

At the time of the audit five tools or approaches had recently been endorsed by WHO. For reference laboratory level, these were liquid TB culture and drug-susceptibility testing, rapid speciation of liquid cultures, and molecular line probe assays (MLPA) for rapid detection of MDR-TB [4], [5]. For peripheral laboratory level, WHO had endorsed the new definition for a smear positive case and the reduction in the number of sputum smears for the diagnosis of smear-positive pulmonary TB [6]–[9]. Two tools were being considered for endorsement by WHO at the time of the survey, namely Light-Emitting Diodes (LED) fluorescence microscopy and the collection of two sputum samples on the same day (called ‘frontloaded’ smear-microscopy) [10]–[12].

Specifically, the audit was designed to answer the following questions:

1. Is information on new tools and the process of retooling getting to NTPs and national reference laboratories (NRLs)?
2. What retooling has taken place recently?
3. What future retooling is planned?
4. What do NTPs consider as constraints of retooling?
5. What do NTPs consider as benefits of retooling?
6. What factors determine whether retooling will take place or not?

The work of the Task Force on Retooling has been mainstreamed and is now the responsibility of the “Introducing New Approaches and Tools” (INAT) Group within the DOTS Expansion Working Group of the Stop TB Partnership. This audit may inform future work of the INAT Group.

## Methods

We set out to conduct an audit of retooling activities in 34 HBCs and/or HBC-MDRs, comprising of nine HBCs, 12 HBC-MDRs and 13 HB- and HBC-MDRs. Questionnaires were sent in March 2009 to 34 NTP managers in all WHO regions (AFRO, AMRO, EMRO, EURO, SEARO and WPRO) with a request to forward the document to their NRL managers.

To address question 1, information on new diagnostics and retooling processes was sent to NTP and NRL managers through electronic files and website links. Their ability to receive and open a large electronic document and publications listed in the “further reading” sections, as well as their ability to access publications through website-based links was assessed.

To address questions 2 and 3, NTP and NRL managers were asked about the status of retooling their NTPs with the seven new diagnostics listed. The results are analyzed distinctively for HBCs and/or HBC-MDRs. To address questions 4, the questionnaires enquired about constraints experienced during introduction of liquid TB culture, rapid speciation tests and MLPA. Since NTP and NRL managers experience the introduction of new tools from different perspectives, their answers are discussed separately.

In addition, all NTP managers who responded to the questionnaire were invited for a telephone interview to collect qualitative data on retooling experiences that were not captured by the questionnaire. NTP managers who agreed to participate were sent information on the pre-determined structure and content of the interview beforehand and their approval was required for the transcripts afterwards. The pre-set open interview questions asked about experienced constraints when adopting the new positive case definition and reducing the number of sputum smears, as well as benefits experienced or anticipated for patients, laboratory staff and the NTP when introducing the seven new diagnostics independently (question 5). An across-case analysis of the interviews was performed to arrive at common concepts. Finally, a with-in case analysis of each individual interview was done to determine possible new considerations for resolving research questions 6.

Ethical approval was not considered necessary for this audit.

## Results

### Respondents

Sixteen out of 34 (47%) HBCs and/or HBC-MDRs responded to the questionnaire, of which four were HBCs, five were HBC-MDRs, and seven were both HB- and HBC-MDRs. Only one country returned two questionnaires filled out by the NTP and NRL manager separately, thus in total 17 questionnaires were collected. The other documents were completed by the NTP manager only (11 countries) or the NRL managers only (four countries). The responding countries represented a wide geographical distribution. Four interviews were conducted through telephone calls with NTP managers from two HBCs in AFRO, one HBC-MDR in EURO and one HB- and HBC-MDR in WPRO.

### Access to information on new tools and retooling processes

Fourteen of the 17 respondents (all except three NTP managers) received and were able to download an electronic document describing the TB diagnostic pipeline. At the end of this document 16 publications for further reading were provided. The ability to access them varied among the questionnaire respondents. Of those that responded, the majority of NTP managers (5/9 or 56%) could open eight documents or more, compared to a minority of NRL managers (1/4 or 25%). These results indicate that NTP managers had better access than NRL heads and could therefore be better informed about new TB diagnostic tools.

Additionally, the respondents were provided with three direct web-based links to WHO documents on TB diagnostic retooling. Fourteen out of 16 respondents (94%, except one NRL heads and one NTP manager) were able to access all three documents. Apparently, it was more effective to provide information through direct web-based linked documents than to have respondents search further readings through a database themselves.

### Retooling under way or completed

The current status of introducing the seven diagnostic tools in NTPs is shown in Table 1. Out of the 16 countries that responded to the questionnaire 10/16 (63%) had implemented liquid culture in their laboratory networks and of those, 9/10 (90%) were using rapid speciation tests. MLPA for drug resistance testing (DRT) had been implemented in 7/16 (44%) HBCs and/or HBC-MDRs, while 8/15 (53%) countries had adopted the new case definition. The recommendation to reduce the number of sputum specimens was implemented in 5/16 (31%) countries.

**Table 1:** Summary of survey results showing 16 high-burden TB (HBCs) and/or high-burden multi-drug resistant TB countries' (HBC-MDRs) perspectives on retooling national TB programmes with seven new TB diagnostic tools.

Health Level	New Tool/Approach	Tools introduced or under way at country level	Constraints experienced/anticipated by NTP and NRL managers	Benefits experienced/anticipated by NTP managers
**Reference laboratory**	**Liquid culture ** ***(endorsed 2007)*** **** [**4**]	Introduced by 10 out of 16 countries: 2 HBCs, 5 HBC-MDRs, 3 HB- and HBC-MDRs. 6 remaining countries all consider adoption	7 NTP managers: low staff capacity, procurement of equipment and reagents, costs, QA system, lab infrastructure, staff training. 3 NRL managers: supply of equipment and reagents, staff training, maintenance, power supply, contamination	4 NTP managers: more rapid detection of MDR-TB, reduced time of diagnosis for patient *(Interviews)*
	**Rapid speciation ** ***(endorsed 2007)*** **** [**4**]	Introduced by 9 out of 16 countries: 2 HBCs, 5 HBC-MDRs, 2 HB- and HBC-MDRs. 7 remaining countries all consider adoption	7 NTP managers: low staff capacity, procurement of equipment and reagents, costs, QA system, lab infrastructure. 2 NRL managers: supply of equipment and reagents, staff training, maintenance	4 NTP managers: rapid detection of MDR-TB, reduced time of diagnosis for patient *(Interviews)*
	**Molecular line-probe assays ** ***(endorsed 2008)*** **** [**5**]	Introduced by 7 out of 16 countries: 1 HBC, 3 HBC-MDRs, 3 HB- and HBC-MDRs. 9 remaining countries all consider adoption	5 NTP managers: lab infrastructure, staff training and capacity, procurement of equipment and reagents, sample transportation. 2 NRL managers: staff training, supply of consumables, power supply	4 NTP managers: more rapid detection of MDR-TB, simultaneous genotyping, separating resistant and sensitive patients *(Interviews)*
**Peripheral laboratory**	**New smear positive case definition ** ***(endorsed 2007)*** **** [**6**], [**7**]	Introduced by 8 out of 15 countries: 3 HBCs, 4 HBC-MDRs, 1 HB- and HBC-MDR. Of 7 remaining countries 4 consider adoption	4 NTP managers: protocol changes, training lab staff, QA system, staff turnover, lack of evidence, overload of recommendations *(Interviews)*	4 NTP managers: reduced time of diagnosis for patient, reduced workload, cost-savings, improved case detection *(Interviews)*
	**Reduction in number of specimens examined ** ***(endorsed 2007)*** **** [**8**], [**9**]	Introduced by 5 out of 16 countries: 3 HBCs, no HBC-MDRs, 2 HB- and HBC-MDRs. Of 10 remaining countries 7 consider adoption	4 NTP managers: protocol changes, sensitizing lab staff, QA system, staff training, lack of evidence, overload of recommendations *(Interviews)*	4 NTP managers: reduced time of diagnosis for patient, reduced workload, cost-savings, improved case detection *(Interviews)*
	**Light-emitting diode-based fluorescence microscopy ** [**10**], [11****]	12 out of 16 countries consider retooling	4 NTP managers: costs of resources, procurement of reagents, convincing staff of benefits, staff training, unsafe reagents, overload of recommendations, lack of expert advise *(Interviews)*	4 NTP managers: reduced examination time, improved case detection, high acceptance among staff, ease of use, cost-savings *(Interviews)*
	**Frontloaded microscopy ** [**12**]	7 out of 16 countries consider retooling	3 NTP managers: training lab staff convincing lab staff of benefits, logistic issues, lack of evidence, increased workload, protocol changes *(Interviews)*	4 NTP managers: reduced time of diagnosis for patient, reduced workload, cost-savings, improved case detection *(Interviews)*

**Legend.** TB: tuberculosis. NTP: national TB programme. NRL: national TB reference laboratory. QA: quality assurance. Results were obtained through questionnaires (and interviews where indicated).

All respondents that had not implemented liquid culture (6/6) planned to do so in the future. The same applied to countries not using rapid methods for speciation (7/7) and MLPA (9/9). More than half of HBCs and/or HBC-MDRs had plans to adopt microscopy-optimizing tools (4/7 or 57% considered the new case definition, 7/11 or 64% a reduction of the number of specimens and 12/16 or 75% LED-fluorescence microscopy (FM)). Frontloaded microscopy appeared a little less appealing; 7/16 (44%) consider implementation. No apparent difference was seen between HBCs and HBC-MDRs in wishing to adopt microscopy-improving diagnostics (data not shown). Most countries wishing to adopt frontloaded and LED-FM would only do so with official WHO endorsement (5/6 or 83% and 9/12 or 75% respectively, data not shown).

### Constraints experienced and anticipated

The questionnaires enquired about the constraints respondents experienced during implementation of three tools, namely liquid culture, rapid speciation tests, and MLPA for DRT. Table 1 shows all responses. The NTP managers that had introduced liquid culture in their countries most often reported low staff capacity and the supply of equipment and consumables into the country as obstacles for adopting the tool. The latter obstacle was also reported most frequently by NRL managers. As constraints of implementing rapid speciation tests, again low personnel capacity and procurement of equipment are mostly mentioned by NTP managers, while NRL managers note supplies, maintenance of equipment and staff training. For adoption of MLPA's, NTP managers reported laboratory infrastructure to be the most important obstacle, and supplies, staff training and uninterrupted electrical power supply were mentioned by NRL managers.

The interviews explored the experienced constraints four NTP managers during the introduction of microscopy improving tools. For the new case definition, respondents mostly mentioned protocol changes and training laboratory staff as obstacles to implementation. For the reduction of sputum samples, most of them identified protocol changes and convincing laboratory staff of the benefits as problems. Additionally, the interviews shed light on the anticipated constraints faced by these NTP managers during future implementation of LED-FM and frontloaded microscopy. The overall costs of resources for LED-FM and the training and convincing of laboratory personnel required to adopt frontloaded microscopy were considered major obstacles.

### Benefits experienced and anticipated

From the interviews with four NTP managers it came forward that the most important experienced benefit of adopting liquid culture, rapid speciation and MLPA in NTPs was considered to be more rapid identification of MDR-TB. All responses are shown in Table 1. Furthermore, the majority of respondents believed that a reduced time of diagnosis for the patient was a benefit of adopting the new case definition and reducing the number of sputum specimen. The advantages of LED-FM and front-loaded microscopy experienced by the respondents were also reduced time of diagnosis for the patient, as well as reduced time of examination for the technicians.

### Additional experiences/opinions gathered through interviews

The with-in case analysis highlighted an interesting concept repeated by one respondent and illustrated by the following quote: *“For me as the manager who initiates these WHO recommended strategies there should be very serious advantages for changing something. (…) It should be one package of recommendations, not just recommend today one thing and tomorrow another thing. This is the reason why I don't initiate this change.”*

This respondent highlighted the difficulty for NTPs of repeatedly dealing with a large number of new retooling recommendations from WHO.

Additionally, respondents believed that other general challenges had to be overcome before successful retooling could take place, especially the establishment of a functional external quality assessment system for microscopy and the enhancement of public access to diagnostic services.

## Discussion

This survey has shed light on NTP managers' access to information on new diagnostic tools and retooling. It appears that information is accessible when provided through a direct web-based link. Documents referenced or recommended for further reading should be open-access or otherwise available through a website. The New Diagnostics Working Group has recently developed a site (www.tbevidence.org) that may serve this purpose [13]. Secondly, the audit has mapped the progress of uptake of new TB diagnostic policies in HBCs and/or HBC-MDRs. About half of these countries have adopted the diagnostic tools newly recommended by the WHO, except the reduction of the number of patient sputum specimens to be examined by microscopy. More advocacy may be needed if this last approach is to be adopted by countries. The survey also demonstrated that implementers are more disposed to adopting modern, technically demanding diagnostic techniques than approaches to optimize smear microscopy, though the latter is likely to remain the cornerstone of TB diagnosis in HBCs for some considerable time. The results suggest NTP staff feel that the introduction of frontloaded microscopy in particular requires the provision of more field evidence and information on specific advantages to NTPs. WHO endorsement of any diagnostic tool was reported as critical in the decision to introduce it to NTP activities. Since the audit was conducted the evidence for LED-based fluorescence microscopy and frontloaded microscopy has been systematically reviewed and evaluated by a WHO-convened Expert Group Meeting. Both of these diagnostic tools have now been endorsed by WHO [14], [15].

The results ultimately illustrated that there is uncertainty among some NTP staff on how to implement multiple recommendations on new diagnostics from WHO. NTPs are required to introduce new tools in a structured manner, often through a multi-year plan. It may be helpful to offer a single recommendation covering the introduction of a package of new diagnostic techniques and approaches appropriate for particular settings. Donors, policy-makers and technical agencies may find aspects of the NTP perspective useful in supporting retooling activities at country level.

This audit has addressed the opportunities and challenges encountered in the process of implementing new diagnostics in NTPs in a subjective manner. The sample size was small with 16 out of 34 HBCs and/or HBC-MDRs responding to the questionnaire and four NTP managers were interviewed. The non-responsive countries could have had different opinions about the benefits and obstacles related to introducing new tools. Therefore, surveys with a broader scope and more objective indicators are required to prioritize these opinions and make sound assumptions about (cost-effective) benefits of introducing new tools. Finally, future studies assessing the success of diagnostic retooling should not only explore the adoption of new tools by NTPs, but also their integration into national TB control activities.
